# Normalization and microbial differential abundance strategies depend upon data characteristics

**DOI:** 10.1186/s40168-017-0237-y

**Published:** 2017-03-03

**Authors:** Sophie Weiss, Zhenjiang Zech Xu, Shyamal Peddada, Amnon Amir, Kyle Bittinger, Antonio Gonzalez, Catherine Lozupone, Jesse R. Zaneveld, Yoshiki Vázquez-Baeza, Amanda Birmingham, Embriette R. Hyde, Rob Knight

**Affiliations:** 1 Department of Chemical and Biological Engineering, University of Colorado at Boulder, Boulder, CO 80309 USA; 2 Departments of Pediatrics, University of California San Diego, 9500 Gilman Drive, MC 0763, La Jolla, CA 92093 USA; 3 Biostatistics and Computational Biology Branch, NIEHS, NIH, Research Triangle Park Durham, NC USA; 4 Department of Microbiology, University of Pennsylvania, Philadelphia, PA 18014 USA; 5 Department of Medicine, University of Colorado, Denver, CO 80204 USA; 6 Department of Microbiology, Oregon State University, 226 Nash Hall, Corvallis, OR 97331 USA; 7 Department of Computer Science & Engineering, University of California San Diego, La Jolla, CA 92093 USA; 8 Center for Computational Biology and Bioinformatics, Dept. of Medicine, University of California San Diego, La Jolla, CA 92093 USA; 9 Center for Microbiome Innovation, University of California San Diego, La Jolla, CA 92093 USA

**Keywords:** Microbiome, Normalization, Differential abundance, Statistics

## Abstract

**Background:**

Data from 16S ribosomal RNA (rRNA) amplicon sequencing present challenges to ecological and statistical interpretation. In particular, library sizes often vary over several ranges of magnitude, and the data contains many zeros. Although we are typically interested in comparing relative abundance of taxa in the ecosystem of two or more groups, we can only measure the taxon relative abundance in specimens obtained from the ecosystems. Because the comparison of taxon relative abundance in the specimen is not equivalent to the comparison of taxon relative abundance in the ecosystems, this presents a special challenge. Second, because the relative abundance of taxa in the specimen (as well as in the ecosystem) sum to 1, these are compositional data. Because the compositional data are constrained by the simplex (sum to 1) and are not unconstrained in the Euclidean space, many standard methods of analysis are not applicable. Here, we evaluate how these challenges impact the performance of existing normalization methods and differential abundance analyses.

**Results:**

Effects on normalization: Most normalization methods enable successful clustering of samples according to biological origin when the groups differ substantially in their overall microbial composition. Rarefying more clearly clusters samples according to biological origin than other normalization techniques do for ordination metrics based on presence or absence. Alternate normalization measures are potentially vulnerable to artifacts due to library size.

Effects on differential abundance testing: We build on a previous work to evaluate seven proposed statistical methods using rarefied as well as raw data. Our simulation studies suggest that the false discovery rates of many differential abundance-testing methods are not increased by rarefying itself, although of course rarefying results in a loss of sensitivity due to elimination of a portion of available data. For groups with large (~10×) differences in the average library size, rarefying lowers the false discovery rate. DESeq2, without addition of a constant, increased sensitivity on smaller datasets (<20 samples per group) but tends towards a higher false discovery rate with more samples, very uneven (~10×) library sizes, and/or compositional effects. For drawing inferences regarding taxon abundance in the ecosystem, analysis of composition of microbiomes (ANCOM) is not only very sensitive (for >20 samples per group) but also critically the only method tested that has a good control of false discovery rate.

**Conclusions:**

These findings guide which normalization and differential abundance techniques to use based on the data characteristics of a given study.

**Electronic supplementary material:**

The online version of this article (doi:10.1186/s40168-017-0237-y) contains supplementary material, which is available to authorized users.

## Background

Although data produced by high-throughput sequencing has been proven extremely useful for understanding microbial communities, the interpretation of these data is complicated by several statistical challenges. Following initial quality control steps to account for errors in the sequencing process, microbial community sequencing data is typically organized into large matrices where the columns represent samples, and the rows contain observed counts of clustered sequences commonly known as operational taxonomic units, or OTUs, that represent bacteria types. These tables are often referred to as OTU tables. Several features of OTU tables can cause erroneous results in downstream analyses if unaddressed. First, the microbial community in each biological sample may be represented by very different numbers of sequences (i.e., library sizes), reflecting differential efficiency of the sequencing process rather than true biological variation. This problem is exacerbated by the observation that the full range of species is rarely saturated, so that more bacterial species are observed with more sequencing (similar trends by sequencing depth hold for discovery of genes in shotgun metagenomic samples [1, 2]). Thus, samples with relatively few sequences may have inflated beta (β, or between sample) diversity, since authentically shared OTUs are erroneously scored as unique to samples with more sequences [3]. Second, most OTU tables are sparse, meaning that they contain a high proportion of zero counts (~90%) [4]. This sparsity implies that the counts of rare OTUs are uncertain, since they are at the limit of sequencing detection ability when there are many sequences per sample (i.e., large library size) and are undetectable when there are few sequences per sample. Third, the total number of reads obtained for a sample does not reflect the absolute number of microbes present, since the sample is just a fraction of the original environment. Since the relative abundances sum to 1 and are non-negative, the relative abundances represent compositional data [5–7]. Compositional data are constrained by the simplex (sum to 1) and are not free floating in the Euclidean space; therefore, standard methods of analysis are not applicable. For example, an increase in abundance of one prevalent bacterial taxon can lead to spurious negative correlations for the abundance of other taxa [8]. Uneven sampling depth, sparsity, and the fact that researchers are interested in drawing inferences on taxon abundance in the ecosystem using the specimen level data represent serious challenges for interpreting data from microbial survey studies.

In an attempt to mitigate some of these three challenges and aid in data interpretation, data are often normalized by various computational processes prior to downstream analysis. Normalization is the process of transforming the data in order to enable accurate comparison of statistics from different measurements by eliminating artifactual biases in the original measurements. For example, in microbiome data, biases that reflect no true difference in underlying biology can exist due to variations in sample collection, library preparation, and/or sequencing and can manifest as, e.g., uneven sampling depth and sparsity. After effective normalization, data from different samples can then be compared to each other. Ordination analysis, such as principal coordinate analysis (PCoA) [9], is often then applied to these normalized data to visualize broad trends of how similar or different bacterial populations are in certain sample types, such as healthy vs. sick patients (ordination is a general term for a family of techniques that summarize and project multivariate community data into lower-dimension space). This enables easy visual inspection of sample groupings, driven by sample bacterial content similarity/dissimilarity, and any association with sample metadata. Researchers may further wish to determine, through statistical testing, which specific bacteria are significantly differentially abundant between two ecosystems; this process is known as differential abundance testing. Significant changes in certain bacterial species abundances are linked to inflammatory bowel diseases [10], diarrhea [11], obesity [12–14], HIV [15], diet [16], culture, age, and antibiotic use [17], among many other conditions. However, the reliability of these findings depends upon how much the statistical challenges posed by the underlying community sequence data impact the chosen normalization and differential abundance testing techniques.

This paper therefore examines how various normalization and differential abundance testing procedures available in the literature are affected by the challenges inherent in microbiome data. Recent work in this area [18] addresses the performance of parametric normalization and differential abundance testing approaches for microbial ecology studies, but it is primarily focused on estimating proportions. We update and expand those findings using both real and simulated datasets exemplifying the challenges noted above.

### Normalization approaches

Because normalization is intended to enable meaningful comparison of data from different measurements, it is critical to the validity of all downstream analyses. Microbial ecologists in the era of high-throughput sequencing have commonly normalized their OTU matrices by rarefying or drawing without replacement from each sample such that all samples have the same number of total counts. This process, in effect, standardizes the library size across samples, mitigating the first challenge discussed above. Samples with total counts below the defined threshold are excluded, sometimes leading researchers to face difficult trade-offs between sampling depth and the number of samples evaluated. To ensure an informative total sum is chosen, rarefaction curves can be constructed [19]. These curves plot the number of counts sampled (rarefaction depth) vs. the expected value of species diversity. Rarefaction curves provide guidance that allows users to avoid gutting the species diversity found in samples by choosing too low a rarefaction depth. The origins of rarefying sample counts are mainly in-sample species diversity measures or alpha diversity [19, 20]. However, more recently rarefying has been used in the context of β-diversity [21, 22]. Rarefying samples for normalization is now the standard in microbial ecology and is present in all major data analysis toolkits for this field [23–26]. While rarefying is not an ideal normalization method, as it potentially reduces statistical power depending upon how much data is removed and does not address the challenge of compositional data, alternatives to rarefying have not been sufficiently developed until recently.

Another common normalization method besides rarefying is scaling. Scaling refers to multiplying the matrix counts by fixed values or proportions, i.e., scale factors, and specific effects of scaling methods depend on the scaling factors chosen and how they are applied. Often, a particular quantile of the data is used for normalization, but choosing the most effective quantile is difficult [4, 27–30]. Furthermore, while microbiome data are frequently sparse as discussed above, scaling can overestimate or underestimate the prevalence of zero fractions, depending on whether zeros are left in or thrown out of the scaling [8, 31]. This is because putting all samples of varying sampling depth on the same scale ignores the differences in sequencing depth (and therefore resolution of species), caused by differing library sizes between the samples. For example, a rare species having zero counts in a small library size sample can have fractional abundance in a large library size sample (unless further mathematical modeling beyond simple total sum scaling, or proportions, is applied). Scaling can also distort OTU correlations across samples, again due to zeros and differences in sequencing depth [5, 6, 8, 32, 33].

A further alternative is Aitchison’s log-ratio transformation [5], which is applicable to compositional data. However, because the log transformation cannot be applied to zeros (which are often well over half of microbial data counts [4]), sparsity can be problematic for methods that rely on this transformation. One approach to this issue is to replace zeros with a small value, known as a pseudocount [7]. While numerous papers discuss the choice of pseudocount values [34–37], which can influence results, there is no clear consensus on how to choose them. A Bayesian formulation to the problem is available in the literature [38]; however, this formulation assumes a Dirichlet-multinomial framework, which imposes a negative correlation structure on every pair of taxa [7, 39].

### Differential abundance testing methods

For OTU differential abundance testing between groups (e.g., case vs. control), a common approach is to first rarify the count matrix to a fixed depth and then apply a nonparametric test (e.g., the Mann-Whitney/Wilcoxon rank-sum test for tests of two groups; the Kruskal-Wallis test for tests of multiple groups). Nonparametric tests are often preferred because OTU counts are not exactly normally distributed [40]. However, when analyzing relative abundance data, this approach does not account for the fact that the relative abundances are compositional. Also, nonparametric tests such as the Kruskal-Wallis test do not fare well in terms of power when sample size is small and/or the data are sparse [4]. Recently, promising parametric models that make stronger assumptions about the data have been developed in the subfields of transcriptomics (“RNA-Seq”) and metagenomic sequencing. These may additionally be useful for microbial marker gene data [4, 18, 27, 30, 41–44]. Such models have greater power if their assumptions about the data are correct; however, studies of these models on RNA-Seq data have shown that they can yield a high level of false negatives or false positives when relevant assumptions are not valid [45].

These parametric models are composed of a generalized linear model (GLM) that assumes a distribution [46], and the choice of distribution is often debatable [4, 18, 45, 47–53]. To allow for extra Poisson variation in the count data, often the Poisson parameter is modeled by a gamma distribution so that the marginal count distribution is negative binomial (NB) [27, 30, 41]. Although the NB model allows for extra Poisson variation, it does not fit the data well when there are many zeros [4, 49]. Zero-inflated GLMs, the most promising of which is the zero-inflated lognormal, attempt to overcome this limitation [4]. The zero-inflated lognormal tries to address sparsity and unequal sampling depth (library size) by separately modeling “structural” zero counts generated by, e.g., under-sequencing and zeros generated by the biological distribution of taxa, while the non-zero counts are modeled by the lognormal distribution.

## Results and discussion

### Normalization efficacy

**Fig. 1:**
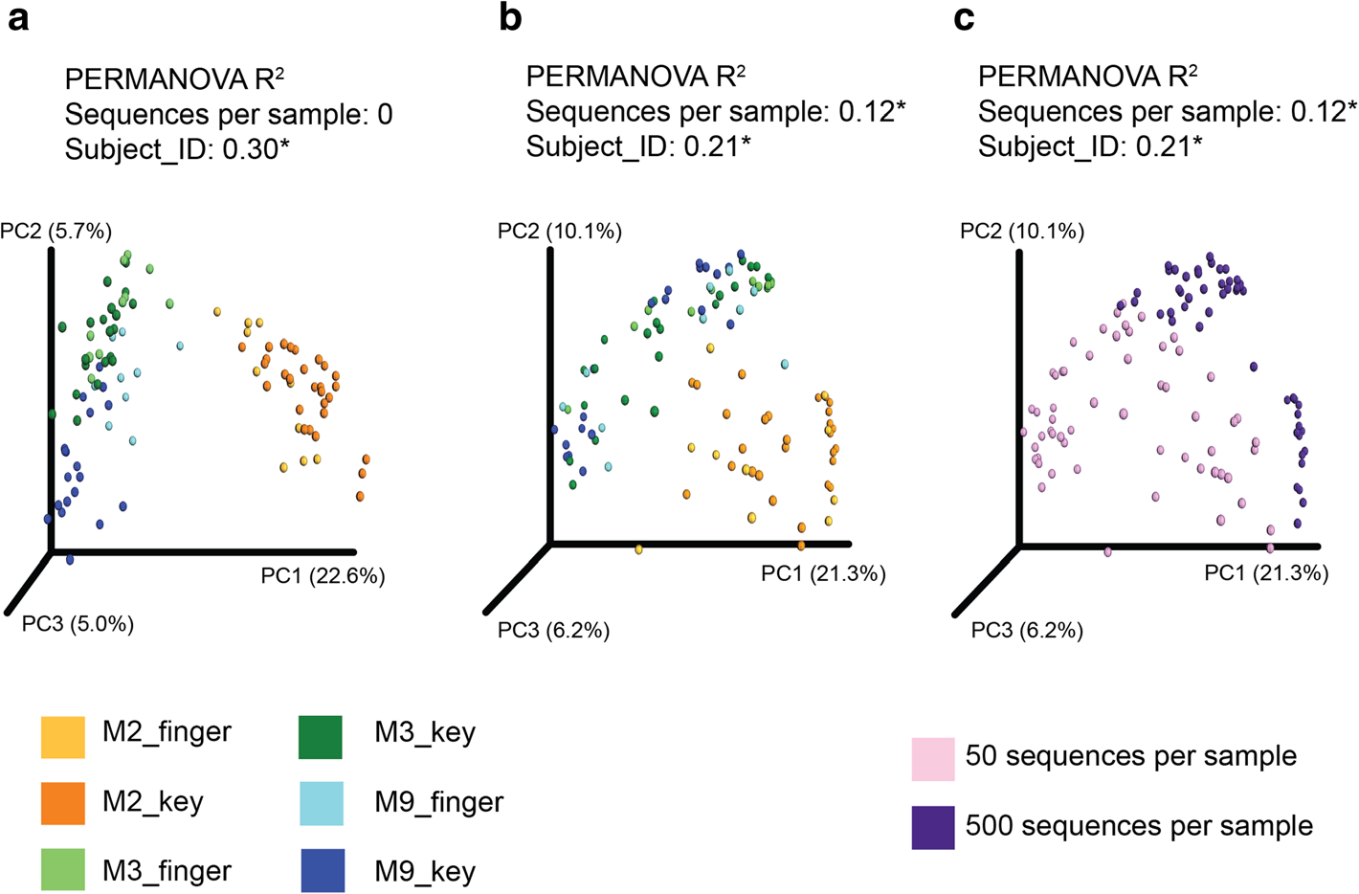
Normalization is critical to result interpretation. **a** The forensic study matching subject’s fingers to the keyboards they touched (Fierer et al.), rarefied at 500 sequences per sample. **b**, **c** Data not normalized, with a random half of the samples subsampled to 500 sequences per sample and the other half to 50 sequences per sample. **b** Colored by subject_ID. **c** Colored by sequences per sample. Nonparametric ANOVA (PERMANOVA) *R* ^2^ roughly represents the percent variance that can be explained by the given variable. *Asterisk* (*) indicates significance at *p* < 0.01. The distance metric of unweighted UniFrac was used for all panels

Proper normalization may potentially remove biases and variations introduced by the sampling and sequencing process, so that the normalized data reflects the underlying biology. One way to evaluate whether normalization has effectively removed such biases is to examine whether the groupings of normalized data after ordination analysis reflect biological or artifactual features. For example, Fig. 1a shows a PCoA plot based on normalized data, which demonstrates that the subjects are matched to the keyboard they touched, and samples from the same subject are close together in PCoA space [54]. With the UniFrac distance metric used here, such close sample grouping indicates similar bacterial communities. However, the grouping becomes less distinguishable when the data is not normalized (Fig. 1b), and this is potentially caused by the artifact of sequence depth variation (Fig. 1c). The highly sequenced samples appear more similar to each other than the shallowly sequenced samples because the highly sequenced samples are scored as sharing the same rare taxa (it should be noted that when groupings in a PCoA plot do not appear to reflect artifactual features, this does not necessarily indicate that they reflect relevant biology, because it is trivial to generate data with no biological signal that nonetheless form or appear to form clusters).

**Fig. 2:**
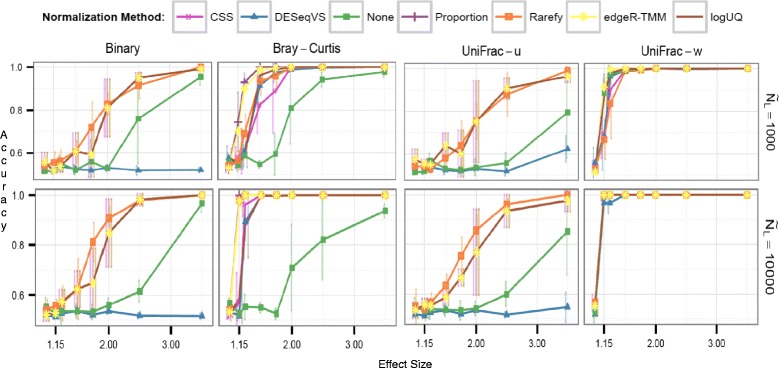
Comparison of common distance metrics and normalization methods across library sizes. Clustering accuracy, or fraction of samples correctly clustered, is shown for all combinations of four common distance metrics (panels arranged from *left to right*) across two library depths (panels arranged from *top to bottom*; N_L_, median library size), six sample normalization methods (series within each panel), and several effect sizes (x-axis within panels). For all methods, samples below the 15th percentile of library size were dropped from the analysis to isolate the effects of rarefying. The x-axis (effect size) within each panel represents the multinomial mixing proportions of the two sample classes *Ocean* and *Feces*. A higher effect size represents an easier clustering task

To assess the seven proposed normalization methods shown in Table 1, we first examined prior simulations [18]. Briefly, only necessary modifications (“Methods” section) were made to the code of McMurdie and Holmes [18], making our approach easily comparable. If all techniques are run on the same samples as those used when rarefying, the rarefying technique clusters as many samples into their biological groupings as the alternatives (Fig. 2). If low-depth samples are not dropped from analysis for methods other than rarefying, this could impact clustering by biological origin, especially for presence/absence distance metrics (Additional file 1: Figure 1S). This practice of removing low-depth samples from the analysis is supported by the recent discovery that small biomass samples are of poorer quality and contain a higher proportion of contaminating sequences [55, 56]. Furthermore, alternatives to rarefying also recommend discarding low-depth samples, particularly if they cluster separately from the rest of the data [4, 42]. These results demonstrate that previous microbiome ordinations using rarefying as a normalization method likely clustered similarly compared to newer techniques, especially if some low-depth samples were removed.

**Table 1:** Normalization methods investigated in this study

Method	Description
None	No correction for unequal library sizes is applied
Proportion	Counts in each column are scaled by the column’s sum
Rarefy	Each column is subsampled to even depth without replacement (hypergeometric model)
logUQ	Log upper quartile—Each sample is scaled by the 75th percentile of its count distribution; then, the counts are log transformed
CSS	Cumulative sum scaling—This method is similar to logUQ, except that CSS enables a flexible sample distribution-dependent threshold for determining each sample’s quantile divisor. Only the segment of each sample’s count distribution that is relatively invariant across samples is scaled by CSS. This attempts to mitigate the influence of larger count values in the same matrix column
DESeqVS	Variance stabilization (VS)—For each column, a scaling factor for each OTU is calculated as that OTU’s value divided by its geometric mean across all samples. All of the reads for each column are then divided by the median of the scaling factors for that column. The median is chosen to prevent OTUs with large count values from having undue influence on the values of other OTUs. Then, using the scaled counts for all the OTUs and assuming a Negative Binomial (NB) distribution, a mean-variance relation is fit. This adjusts the matrix counts using a log-like transformation in the NB generalized linear model (GLM) such that the variance in an OTU’s counts across samples is approximately independent of its mean
edgeR-TMM	Trimmed Mean by M-Values (TMM)—The TMM scaling factor is calculated as the weighted mean of log-ratios between each pair of samples, after excluding the highest count OTUs and OTUs with the largest log-fold change. This minimizes the log-fold change between samples for most OTUs. The TMM scaling factors are usually around 1, since TMM normalization, like DESeqVS, assumes that the majority of OTUs are not differentially abundant. The normalization factors for each sample are the product of the TMM scaling factor and the original library size

For unweighted metrics that are based on species presence and absence, like binary Jaccard and unweighted UniFrac, the variance-stabilizing transformation performed by DESeq clusters many fewer samples according to biological origin than other techniques. This is because, as done in McMurdie and Holmes [18]), the negative values resulting from the log-like transformation are set to zero, causing the method to ignore many rare species completely. No good solution currently exists for the negative value output by the DESeq technique. DESeq was developed mainly for use with Euclidean metrics [57, 58], for which negative values are not a problem; however, this issue yields misleading results for ecologically useful non-Euclidean measures, like Bray-Curtis [59] dissimilarity. Also, the negative values pose a problem to the branch length of UniFrac [57, 58]. The alternative to setting the negative values to zero, which is adding the absolute value of the lowest negative value back to the normalized matrix, will not work with distance metrics that are not Euclidean because it amounts to multiplying the original matrix by a constant due to the log-like transformation of DESeq [28]. Also, the addition of a constant (or pseudocount; here, one) to the count matrix prior to cumulative sum scaling (CSS) [4], DESeq [30], and logUQ [28] transformation as a way to avoid log(0) is not ideal, because clustering results have been shown to be very sensitive to the choice of pseudocount, due to the nonlinear nature of the log transform [36, 37]. This underscores the need for a better solution to the zero problem so that log-ratio approaches inspired by Aitchison can be used [5] and is especially critical because matrices of microbial counts routinely contain zero values for an overwhelming majority of entries [4]. However, recent work has been done on Bayesian methods for zero estimation, with promising results [38, 60]. Furthermore, DESeq and edgeR-trimmed mean by *M* values (TMM) make the assumptions that most microbes are not differentially abundant, and of those that are, there is an approximately balanced amount of increased/decreased abundance [28]; these assumptions are likely not appropriate for highly diverse microbial environments.

The simulations of Fig. 2 and Additional file 1: Figure S1 are relatively simple—the median library size of the two groups is approximately the same and there is no preferential sequencing. Hence, techniques like no normalization, or sample proportions, do well particularly in weighted UniFrac. It could be argued that if there were preferential sequencing in this simulation, CSS normalization would exhibit superior performance for weighted metrics [4, 36, 37]. It is regrettably beyond the scope of this paper to prove the “correct” normalization technique, but we further examine the unweighted measures.

We next applied the normalization techniques to several datasets from the literature to assess performance in light of the additional complexity inherent to real-world data. To perform an initial, detailed comparison of normalization methods, we selected the data set from Gevers et al. [10]. The data was the largest pediatric Crohn’s disease cohort at the time of publication. The rarefied data was rarefied to 3000 sequences/sample, for all other normalization method samples with fewer than 3000 sequences/sample were removed from the raw data.

**Fig. 3:**
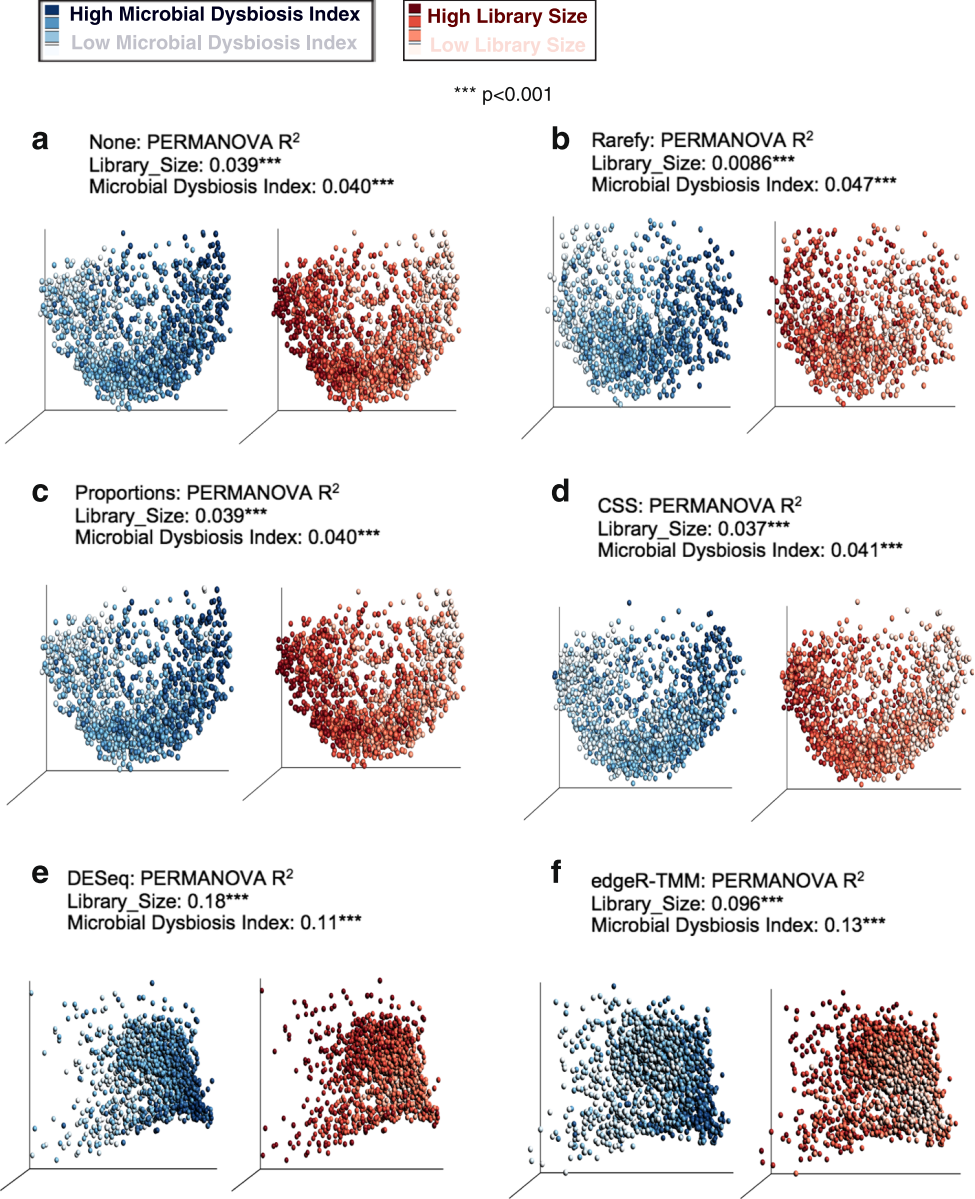
For unweighted distance measures, rarefying diminishes the effect of original library size. Nonparametric multivariate ANOVA (PERMANOVA) was calculated on the Inflammatory Bowel Disease (IBD) dataset of Gevers et al., using type I sequential sums of squares in the linear model (y~Library_Size + Microbial_Dysbiosis_Index). Unweighted UniFrac was used for clustering, except for letters (**e**–**f**) corresponding to weighted UniFrac DESeq and edgeR. The unweighted UniFrac distance matrix for DESeq and edgeR was zero, so a PCoA plot could not be made. For each letter (**a**–**f**), the *left* PCoA plot is colored according to microbial dysbiosis (left legend), and the *right* PCoA plot is colored according to library size (right legend)

Using the data set from Gevers et al. [10], we observed substantial biases/confounding of results due to sequencing depth in PERMANOVA [61], partially because of low biological effect size (*R* ^2^ or sum of squares’ group/total sum of squares) (Fig. 3). In the ordination of unweighted UniFrac distance by PCoA, all normalization methods except rarefying exhibited library size effects of similar *R* ^2^ magnitude as non-normalized data (Fig. 3a). DESeq and edgeR found no difference in the samples using unweighted UniFrac, potentially because of pseudocount addition. Thus, particularly with presence/absence metrics and low effect sizes, proper normalization is critical.

PCoA plots using ecologically common metrics for all of the normalization techniques on a few key real datasets representing a gradient [62], distinct body sites [63], and time series [64] are shown in Additional files 2 and 3: Figures S2–S3. Most normalization methods group according to biology in these cases where there is strong biologic difference. However, some clustering according to depth persists. For example, in the “Moving Pictures of the Human Microbiome” dataset [64], there is secondary clustering by sequence depth within each of the four main clusters when normalization alternatives to rarefying are applied.

Thus, both simulations and real data suggest that rarefying remains a useful technique for sample normalization prior to ordination and clustering for presence/absence distance metrics that have historically been very useful (such as binary Jaccard and unweighted UniFrac [57] distances). Other methods, for weighted distance measures and when sequencing depth is not a confounding variable, are promising. A more thorough study of the effects of compositional data on beta-diversity analysis is needed, as well as better tests for risk of incorrect results due to compositional data; we briefly investigate this topic in the next section.

### Differential abundance testing

Many statistical methods have been proposed in the literature to compare the (relative) taxon abundance between two groups (e.g., case vs. control). Some statistical methods developed specifically for RNA-Seq data, such as DESeq [30], DESeq2 [42], edgeR [27, 44], and Voom [43] (Table 2), have been proposed for use on microbiome data [18] (note that because we found DESeq to perform similarly to DESeq2, except for very slightly lower sensitivity and false discovery rate (FDR), the former is not explicitly included in our results). On the other hand, metagenomeSeq [4, 65] and analysis of composition of microbiomes (ANCOM) [7] were developed specifically for microbial datasets, which usually contain many more zeros than RNA-Seq data. Except for ANCOM, all these approaches incorporate more sensitive statistical tests than the standard nonparametric tests such as the Wilcoxon rank-sum test, and they make some distributional assumptions. Therefore, they may have greater power to detect differentially abundant rare OTUs. Although ANCOM uses the Mann-Whitney test to avoid any distributional assumptions, which are not always easy to verify, its power can be improved by replacing the Mann-Whitney test by a parametric test while making some distributional assumptions.

**Table 2:** Differential abundance methods investigated in this study

Method	Description
Wilcoxon rank-sum test	Also called the Mann-Whitney *U* test. A non-parametric rank test, which is used on the un-normalized (“None”), proportion normalized, and rarefied matrices
DESeq	nbinom Test—a negative binomial model conditioned test. More conservative shrinkage estimates compared to DESeq2, resulting in stricter type I error control
DESeq2	nbinomWald Test—The negative binomial GLM is used to obtain maximum likelihood estimates for an OTU’s log-fold change between two conditions. Then Bayesian shrinkage, using a zero-centered normal distribution as a prior, is used to shrink the log-fold change towards zero for those OTUs of lower mean count and/or with higher dispersion in their count distribution. These shrunken long fold changes are then used with the Wald test for significance
edgeR	exact Test—The same normalization method (in *R*, method = RLE) as DESeq is utilized, and for differential abundance testing also assumes the NB model. The main difference is in the estimation of the dispersion, or variance, term. DESeq estimates a higher variance than edgeR, making it more conservative in calling differentially expressed OTUs
Voom	Variance modeling at the observational level—library sizes are scaled using the edgeR log counts per million (cpm) normalization factors. Then LOWESS (locally weighted regression) is applied to incorporate the mean-variance trend into precision weights for each OTU
metagenomeSeq	fitZIG—a zero-inflated Gaussian (ZIG) where the count distribution is modeled as a mixture of two distributions: a point mass at zero and a normal distribution. Since OTUs are usually sparse, the zero counts are modeled with the former, and the rest of the log transformed counts are modeled as the latter distribution. The parameters for the mixture model are estimated with an expectation-maximization algorithm, which is coupled with a moderated *t* statistic
	fitFeatureModel—a feature-specific zero-inflated lognormal model with empirical Bayes shrinkage of parameter estimates
ANCOM	Analysis of composition of microbiomes—compares the log ratio of the abundance of each taxon to the abundance of all the remaining taxa one at a time. The Mann-Whitney *U* is then calculated on each log ratio

**Fig. 4:**
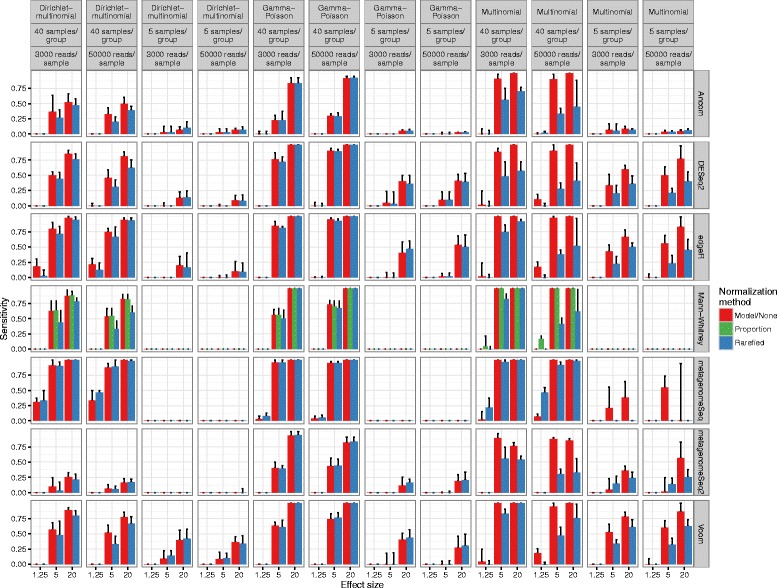
Differential abundance detection sensitivity with varied library sizes, that are approximately even on average between groups. Multinomial, Dirichlet-multinomial, and gamma-Poisson parameters were fit using actual OTU tables from many global environments. *Fold change* represents the fold change of the true positive OTUs from one condition (e.g., case) to another (e.g., control). The *height* of each bar represents the median value from three simulations. Vertical lines extend to the upper quartile of the simulation results. *Model/none* represents data analyzed with a parametric statistical model (e.g., DESeq) or no normalization. *Blue lines* in, e.g., the DESeq row represents the data that was rarefied, then DESeq was applied. Since the fitZIG model depends upon original library size information, the model has high FDR on rarefied data. A 0.01 pseudocount for edgeR was necessary to avoid log(0)

**Fig. 5:**
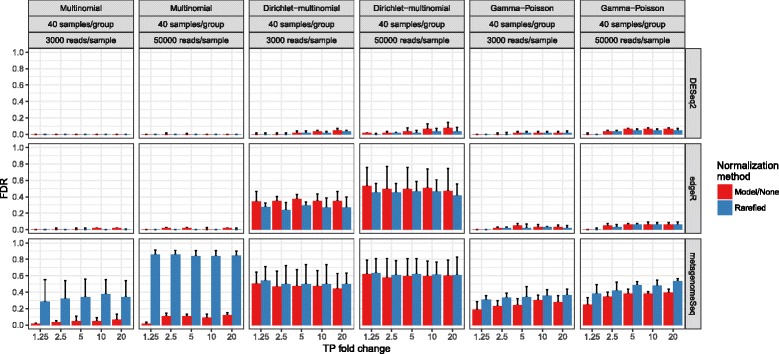
Differential abundance detection false discovery rate with varied library sizes that are approximately even on average between groups. For simplicity, only those methods where the FDR exceeds or is close to 0.05 are shown. Full methods are in Additional file 7: Figure S6. Labels are the same as in Fig. 4

Previous work in this area concluded that the newer differential abundance testing methods are worthwhile, and that the traditional practice of rarefying causes a high rate of false discoveries [18]. However, the latter conclusion was due to an artifact of the simulation (see the “Methods” section; Additional file 4: Figure S4a, Additional file 5: Statistical Supplement A and B). Instead, we found that rarefying itself does not cause a high rate of false discoveries, but rather leads to false negatives (lower sensitivity) driven by the discarding of data and the nonparametric nature of the Wilcoxon rank-sum (Figs. 4 and 5). The severity of the power decrease caused by rarifying depends upon how much data has been thrown away and how many samples were collected. This problem has been known for a long time, leading to the general guideline to rarefy to the highest depth possible [66]. If samples are discarded, justification should be explained.

We recognize that the multinomial and the Dirichlet-multinomial (DM) distributions are not necessarily appropriate for the microbial taxon counts because under such probability distributions, every pair of taxa are negatively correlated, whereas as discussed in Mosimann [39] and Mandal et al. [8], not every pair of OTUs is in fact likely to be negatively correlated. However, because multinomial and DM distributions have been used by several authors [50, 67, 68], we include those distributions in our simulation study for purely comparative purposes. Additionally, we simulate data using the gamma-Poisson [7] (Figs. 4 and 5 and Additional files 6 and 7: Figures S5–S6). As expected, sensitivity increased with library size, but much more so for higher sample sizes, for all methods. Again as expected, for the nonparametric methods and small sample sizes, sensitivity was lower compared to parametric methods. For the parametric methods, in particular fitZIG and edgeR, the underlying data distribution changed results dramatically (Fig. 5 and Additional file 7: Figure S6). This is likely due to each model’s distributional assumptions, e.g., the gamma-Poisson distribution is a closer relative to the negative binomial assumption of edgeR and DESeq; therefore, FDR is lower for the gamma-Poisson vs. the Dirichlet-multinomial. FitZIG was the only method where rarefying increased the FDR, because model parameters require original library size. Also, the 0.05 FDR thresholds were exceeded for techniques like DESeq2 and edgeR with more numbers of samples per group, possibly due to the increased degrees of freedom and decreased shrinkage of dispersion estimates.

**Fig. 6:**
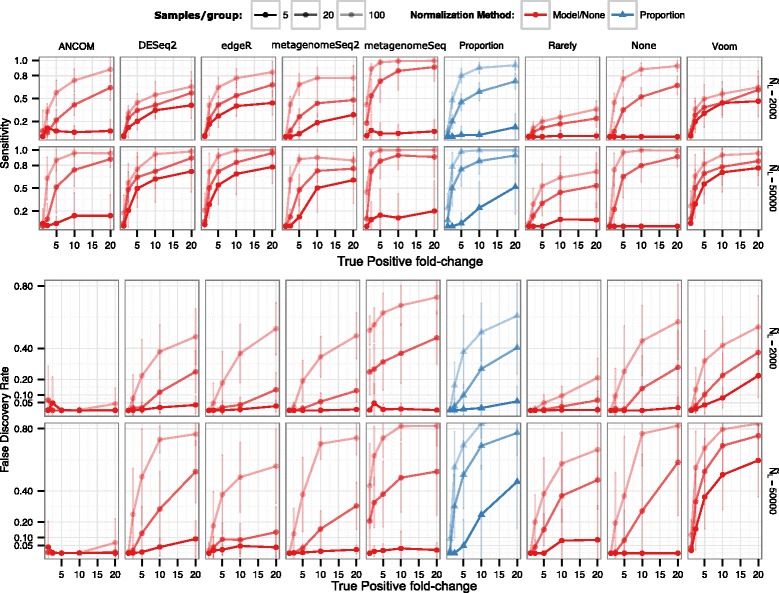
Differential abundance detection performance when sample relative abundances do not reflect ecosystem relative abundances. 10% of OTUs are differentially abundant. For simplicity, only a multinomial model of 2000 OTUs was used, but is the same model as that in Figs. 4 and 5. Labels are the same as in Fig. 4

One of the objectives of a microbiome study is to compare the abundance of taxa in the ecosystem of two or more groups using the observed taxa abundance in specimens drawn from the ecosystem. As noted in Mandal et al. (2015) [7] and in the Additional file 5: Statistical Supplement C, even though the average taxa abundance of a taxon is the same in two ecosystems, it is not necessary that their mean relative abundances are same. Thus, drawing inferences regarding the mean taxon abundance between ecosystems using the specimen level data is a challenging problem. We performed a simulation study to evaluate the performance of various methods in terms of the false discovery rate and power when testing hypotheses regarding mean taxon abundance between ecosystems using the specimen level data. In simulations where the abundances of 10% of the OTUs increased in one group, all but ANCOM [7] had a highly inflated average FDR, in some cases exceeding 40% (Fig. 6). False discovery for all methods increases when the fold change increases, because a larger constant applied to one OTU’s counts impacts the relative abundance of other OTUs. Proportion normalization is known to have high FDR when faced with compositional data [6]. For DESeq/DESeq2, poor performance may be due to the model’s assumption that differentially abundant OTUs are not a large portion of the population [29] or the model’s overdispersion estimates [4]. Because most researchers want to infer ecosystem taxon relative abundances from sampling, this indicates a large previously unsolved problem in differential abundance testing [6].

**Fig. 7:**
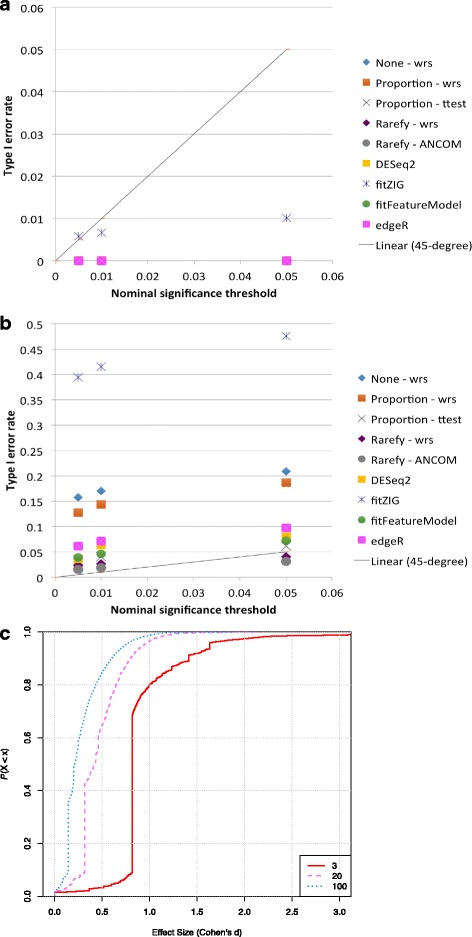
False discovery rate increases when methods are challenged with very uneven library sizes. Real data from one body site was randomly divided into two groups, creating a situation in which there should be no true positives. **a** Uneven library sizes, 3 samples per group. **b** Uneven library sizes, 100 samples per group. For uneven library sizes, the group means differed by 10× (e.g., 40,000 sequences per sample vs. 4000 sequences per sample). The 45-degree line shows where the nominal FDR should equal the observed FDR. **c** Cumulative distribution functions of the effect sizes for 3, 20, and 100 samples per group presented in **a** and **b**. Voom was excluded because it was found to have a higher type I error rate than fitZIG

We also investigated the performance of the techniques on real null data, in which there should be no “true positives,” since samples from the same biological group were randomly divided into two groups (“Methods” section). Most methods, except fitZIG, correctly predict no or very few false positives and are more conservative with decreasing sample size. However, for uneven library sizes and with 20–100 samples per group (Fig. 7a–b), the type 1 error rate of many methods increases beyond the nominal threshold. This was especially so for the no normalization and proportion normalization approaches. We did not observe increased type 1 error with ANCOM. The lack of increased type I error with rarefied data could simply be due to the loss of power resulting from rarefied data [18]. Additionally, manually adding a pseudocount (e.g., one) to the data matrix increases the type 1 error rate beyond the nominal threshold for uneven library sizes (Additional file 8: Figure S7a–b). Instead, one may consider standard zero imputation methods [69]. Interestingly, in the case of very small systematic biases (median effect size <1) as present in the raw data of Fig. 7c, the *t* test on proportion-normalized data outperforms the nonparametric Wilcoxon rank-sum test in Fig. 7. This suggests that in the case of very small systematic biases, rank-based non-parametric tests (except fitZIG) could actually underperform parametric tests, as they do not take into account effect sizes. However, more investigation is necessary.

While the no normalization or proportion approaches control the FDR in cases where the average library size is approximately the same between the two groups (Figs. 4 and 5), they do not when one library is 10× larger than the other (Figs. 3 and 7). Therefore, we reiterate that neither the no normalization nor the sample proportion approach should be used for most statistical analyses. To demonstrate this, we suggest the theoretical example of a data matrix with half the samples derived from diseased patients and half from healthy patients. If the samples from the healthy patients have a 10× larger library size, OTUs of all mean abundance levels will be found to be differentially abundant simply because they may have 10× the number of counts in the healthy patient samples (such systematic bias can happen if, for example, healthy vs. diseased patients are sequenced on separate sequencing runs or are being compared in a meta-analysis). The same warning applies for proportions, especially for rare OTUs that could be deemed differentially abundant because the rare OTUs may not be detected (zero values) in low library size samples, but are non-zero in high library size samples.

**Fig. 8:**
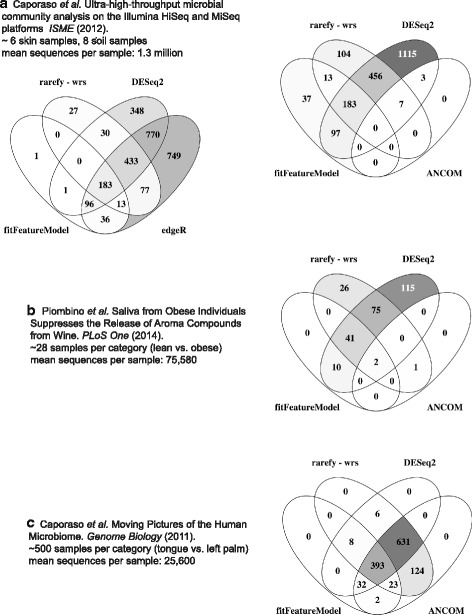
On real datasets, methods disagree especially for few samples per group. FDR *p* < 0.05. *Darker colors* indicate a larger proportion of OTUs discovered by a technique or combination of techniques

Using real data, we further tested the techniques shown to be most promising in the simulations: DESeq2 [42], metagenomeSeq [4, 65], rarefying with the Mann-Whitney U test, and ANCOM [7]. Ranges of dataset sizes were analyzed for environments that likely contain differentially abundant OTUs, as evidenced by the previously published PCoA plots and significance tests (Fig. 8). Six human skin and eight soil samples from Caporaso et al. [70], 28 samples in each of the lean vs. obese groups from Piombino et al. [71], and 500 samples in each of the tongue vs. left palm groups from Caporaso et al. [64] were tested. Although we do not necessarily know which OTUs are true positives in these actual data, it is of interest to investigate how the most promising techniques compare to each other. edgeR, which is known to underestimate dispersion, resulting in a high FDR [4, 42, 45, 53], predicts an extremely large number of significantly differentially abundant OTUs relative to other methods, especially for studies with small sample sizes (Fig. 8a). Additionally, in Fig. 8a, DESeq and edgeR predict well over half the OTUs to be differentially abundant, a violation of the associated normalization assumptions of constant abundance of a majority of species. While the disagreement in significantly differentially abundant OTU predictions decreases with increased library size, there is concern that simulations, no matter how carefully constructed, cannot mimic the complexity of real microbiome data.

## Conclusions

We confirm that recently developed more complex techniques for normalization and differential abundance testing hold potential. Of methods for normalizing microbial data for ordination analysis, we found that DESeq normalization [30, 42], which was developed for RNA-Seq data and makes use of a log-like transformation, does not work well with ecologically useful metrics, except weighted UniFrac [58]. DESeq normalization requires more development for general use on microbiome data. With techniques other than rarefying, library size is a frequent confounding factor that obscures biologically meaningful results. This is especially true with very low library sizes (under approximately 1000 sequences per sample) or if presence/absence metrics like unweighted UniFrac are used [57]. Additionally, many microbial environments are extremely variable in microbial composition, which would violate DESeq and edgeR-TMM normalization assumptions of a constant abundance of a majority of species and of a balance of increased/decreased abundance for those species that do change. Therefore, rarefying is still a useful normalization technique: rarefying can more effectively mitigate the artifact of sample library size than other normalization techniques and results in a higher PERMANOVA *R* ^2^ for the studied biological effect, especially for small (<1000 sequences per sample) and very uneven (>~10× on average) library sizes between groups. The approaches of no normalization and sample proportion are prone to generation of artifactual clusters based on sequencing depth in beta diversity analysis. Therefore, researchers should proceed with caution and check for these effects in ordination results if the count data was not rarefied. In PERMANOVA tests, we recommend that a term for library size is included if the data set was not rarefied or otherwise normalized.

For differential abundance testing, we used both simulations and real data. Overall, we found that simulation results are very dependent upon simulation design and distribution, highlighting the need for gold standard datasets. We confirm that techniques based on GLMs with the negative binomial or log-ratios are promising. DESeq2 [42] was designed for, and provides, increased sensitivity on smaller datasets (<20 samples per group); however, it tends towards a higher false discovery rate with larger and/or very uneven library sizes (>~10× on average). The practice of manually adding a pseudocount to the matrix prior to DESeq2 transformation increases the FDR. This agrees with prior investigation finding RNA-Seq approaches unsuitable for microbiome data [60]. If the average library size for each group is approximately equal, then rarefying itself does not increase the false discovery rate. For groups with large (~10×) differences in the average library size between groups, rarefying helps decrease the false discovery rate. Prior to analysis, researchers should assess the difference in average library size between groups. If large variability in library sizes across samples is observed, then rarefying is useful as a method of normalization. ANCOM [7] maintains a low FDR for all sample sizes and is the only method that is suitable for making inferences regarding the taxon abundance (as well as the relative abundance) in the ecosystem using the abundance data from specimens. With ANCOM, sensitivity is decreased on small datasets (<20 samples per group), partially due to its use of the Mann-Whitney test. ANCOM with more sensitive statistical tests needs to be investigated.

Thanks to McMurdie and Holmes’ previous work in this area [18], we recognize the potential of these newer techniques and have incorporated DESeq2 [42] and metagenomeSeq [4, 65] normalization and differential abundance testing into QIIME version 1.9.0 [24], along with the traditional rarefying and non-parametric testing techniques. ANCOM differential abundance testing is included in scikit-bio (scikit-bio.org) and will be part of QIIME version 2.0.

## Methods

### Normalization

The basic test of how well broad differences in microbial sample composition are detected, as assessed by clustering analysis, was conducted in “simulation A” from McMurdie and Holmes [18]. Briefly, the “ocean” and “feces” microbiomes (the microbial data from ocean and human feces samples, respectively) from the “Global Patterns” dataset [72] were used as templates, modeled with a multinomial, and taken to represent distinct classes of microbial community because they have few OTUs in common. These two classes were mixed in eight defined proportions (the “effect size”) in independent simulations in order to generate simulated samples of varying clustering difficulty. Samples were generated in sets of 40, as in McMurdie and Holmes [18]. We also tested smaller and larger sample sizes but saw little difference in downstream results. Additional sets of 40 samples were simulated for varying library sizes (1000, 2000, 5000, and 10,000 sequences per sample). These simulated samples, done in triplicate for each combination of parameters, were then used to assess normalization methods by the proportion of samples correctly classified into the two clusters by the partitioning around medioids (PAM) algorithm [73, 74].

McMurdie and Holmes [18] evaluated clustering accuracy with five normalization methods (none, proportion, rarefying with replacement as in the multinomial model [75], DESeqVS [30], and UQ-logFC (in the edgeR package) [27]) and six beta-diversity metrics (Euclidean, Bray-Curtis [59], PoissonDist [76], top-MSD [27], unweighed UniFrac [57], and weighted UniFrac [58]). We modified the normalization methods to those in Table 1 (none, proportion, rarefying without replacement as in the hypergeometric model [75], CSS [4], logUQ [28], DESeqVS [30], and edgeR-TMM [27]) and the beta diversity metrics to those in Fig. 2 and Additional file 1: Figure S1 (binary Jaccard, Bray-Curtis [59], Euclidean, unweighed UniFrac [57], and weighted UniFrac [58]), thus including more recent normalization methods [4, 28] and only those beta diversity metrics that are most common in the literature. We amended the rarefying method to the hypergeometric model [75], which is much more common in microbiome studies [23, 24]. Negative values in the DESeq normalized values [30] were set to zero as in McMurdie and Holmes [18], and a pseudocount of one was added to the count tables [18]. McMurdie and Holmes [18] penalized the rarefying technique for dropping the lowest fifteenth percentile of sample library sizes in their simulations by counting the dropped samples as “incorrectly clustered.” Because the 15th percentile was used to set rarefaction depth, this capped clustering accuracy at 85%. We instead quantified cluster accuracy among samples that were clustered following normalization to exclude this rarefying penalty (Fig. 2). Conversely, it has since been confirmed that low-depth samples contain a higher proportion of contaminants (rRNA not from the intended sample) [55, 56]. Because the higher depth samples that rarefying keeps may be higher quality and therefore give rarefying an unfair advantage, Additional file 1: Figure S1 compares clustering accuracy for all the techniques based on the same set of samples remaining in the rarefied dataset.

On the real datasets, nonparametric multivariate ANOVA (PERMANOVA) [77] was calculated by fitting a type I sequential sums of squares in the linear model (y~Library_Size + Biological_Effect). Thus, we control for library size differences before assessing the effects on the studied biological effect. All data was retrieved from QIITA (https://qiita.ucsd.edu/).

### Differential abundance testing

#### Multinomial distribution

The simulation test for how well truly differentially abundant OTUs are recognized by various parametric and nonparametric tests was conducted as in “simulation B” in McMurdie and Holmes [18], with a few changes. The basic data generation model remained the same, but the creation of true positive OTUs was either made symmetrical through duplication or moved to a different step, so that the OTU environmental abundances matched their relative abundances. The “Global Patterns” [72] dataset was again used, because it was one of the first studies to apply high-throughput sequencing to a broad range of environments, which includes nine environment types from “ocean” to “soil”; all simulations were evaluated for all environments. Additionally, we verified the results on the “lean” and “obese” microbiomes from a different study [71]. As in McMurdie and Holmes, correction for multiple testing was performed using the Benjamini & Hochberg [78] FDR threshold of 0.05.

A simple overview of the two methods used for simulating differential abundance is presented in Additional file 4: Figure S4a. In McMurdie and Holmes’ [18] “original” simulation (second row), the distribution of counts from one environment (e.g., ocean) was modeled off of a multinomial template (first row) for two similar groups (ocean_1 and ocean_2), ensuring a baseline of all “true negative” OTUs. Following the artificial inflation of specific OTUs in the ocean_1 samples to create true positives, fold-change estimates for every other OTU are affected. Thus, although in terms of abundances, their set-up allows for some true positives and true negatives, in terms of relative abundances, by their sampling scheme, some taxa are true positives. Thus, true negatives are possible true positives in terms of relative abundances. To ensure that not all taxa are true positives in terms of relative abundances, we created pairs of differentially abundant OTUs in both the ocean_1 and ocean_2 samples (third row), and thus created a new “balanced” simulation where the same taxa are differentially abundant as well as differentially relative abundant. Details are in Additional file 5: Statistical Supplement A and B.

However, in general, as noted in Additional file 5: Statistical Supplement C, equality of taxa abundance between two environments does not translate to equality of the relative abundance of taxa between two environments. In terms of statistical tests, depending upon what parameter is being tested, this can result in inflated false discovery rates. To illustrate this phenomenon, we conducted a simulation study mimicking Additional file 4: Figure S4b, with results in Fig. 6, where two environments had differentially abundant taxa. Samples were generated from such environments according to a multinomial distribution and these specimen level data were used to compare the taxa abundance in the two environments.

Besides the above procedural changes to the McMurdie and Holmes [18] simulation, we also modified the rarefying technique from sampling with replacement (multinomial) to sampling without replacement (hypergeometric—as in the previous normalization simulations) [75]. The testing technique was modified from a two-sided Welch *t* test to the nonparametric Mann-Whitney test, which is widely used and more appropriate because the OTU distributions in microbiome data usually deviate from normality. The techniques used (Table 2) differ only by the addition of another metagenomeSeq method, fitFeatureModel [65], another RNA-Seq method, Voom [43], and ANCOM [8]. This new simulation code, for which all intermediate files and dependencies are easily available, can be found in the supplemental R files (Additional file 9 and 10).

#### Dirichlet-multinomial distribution

This simulation was exactly the same as the above “multinomial” simulation, except that the Dirichlet-multinomial distribution was used instead of the multinomial distribution to model the nine environments found in the Global Patterns [72] dataset. Dirichlet-multinomial was a better fit for the Global Patterns data, as determined through the “HMP” package [67] and the C(α)-optimal test-statistics [79]. Dirichlet-multinomial parameters were calculated through the method of moments estimators. As a check, we ensured that the Dirichlet-multinomial results converged to the multinomial results with large gamma.

#### Gamma-Poisson distribution

This simulation was exactly the same as the above multinomial simulation, except that the gamma-Poisson distribution was used instead of the multinomial distribution to model the nine environments found in the Global Patterns [72] dataset. The means and the variances of the OTUs across samples for each of the environments were used to estimate the lambdas of the gamma-Poisson distribution. As a check, we ensured that the gamma-Poisson results converged to the multinomial results with large shape parameter.

#### Real null data

For the experimental, null data test, we selected random samples from within one body site (“left hand”) of Caporaso et al. [64]. We then randomly divided the samples into two groups, each having 3, 20, and 100 samples, and applied the differential abundance methods. For the case where the library sizes between the two groups differed by ~10×, we selected some of the lowest and highest library size samples within the left hand group.

#### ANCOM

The ANCOM procedure compares the relative abundance of a taxon between two ecosystems by computing Aitchison’s [5] log-ratio of abundance of each taxon relative to the abundance of all remaining taxa one at a time. Thus, if there are “m” taxa, then for each taxon it performs “m-1” tests and the significance of each test is determined using the Benjamini-Hochberg procedure that controls for FDR at 0.05. For each taxon, ANCOM counts the number of tests among the m-1 tests that are rejected. Thus for each taxon, ANCOM obtains a count random variable *W* that represents the number of nulls among the m-1 tests that are rejected. ANCOM determines the final significance of a taxon by using the empirical distribution of *W*. To deal with zero counts, we use an arbitrary pseudo count value of 0.001. For a more detailed description of ANCOM, we refer the reader to Mandal et al. [7].

## Additional files

Additional file 1: Figure S1. Comparison of common distance metrics and normalization methods when low-coverage samples are excluded. The right axis represents the median library size (N_L_), while the x-axis effect size is the multinomial mixing proportions of the two classes of samples, *ocean* and *feces*. For rarefying, samples below the 15th percentile of library size were dropped from the analysis. See caption for Fig. 2 for further details. (PDF 432 kb)
Additional file 2: Figure S2. All normalization techniques on key microbiome datasets, Bray Curtis distance. *Rows* of panels show (from *top to bottom*) data from 88 soils [62], body sites [63], and moving pictures [64]. 88 soils are colored according to a color gradient from low to high pH. The Costello et al. body sites’ dataset is colored according to body site feces (*blue*) and oral cavity (*purple*); the rest of the colors are external auditory canal, hair, nostril, skin, and urine. Moving pictures dataset: left and right palm (*red*/*blue*), tongue (*green*), and feces (*orange*). It is important to note that all the samples in these datasets are approximately the same depth, and there are very strong driving gradients. (PNG 1357 kb)
Additional file 3: Figure S3. All normalization techniques on key microbiome datasets, unweighted UniFrac distance. See Figure S3 caption for details. (PNG 1368 kb)
Additional file 4: Figure S4. Simple example of the reasoning behind differential abundance simulations. **a** In actual OTU tables generated from sequencing data, the counts (*left column*) are already compositional and therefore only relative. Application of a constant multiplier to the original multinomial template to create fold-change differences disturbs the distinction between true positive (TP) and true negative (TN) OTUs in the original simulation, but not the balanced simulation. **b** Creation of compositional data from the multinomial template. The sum constraint that occurs when a researcher samples from the environment can cause, e.g., unchanged OTUs to appear changed in an OTU table. (PDF 199 kb)
Additional file 5: Statistical supplement: statistical details of simulation studies. (DOCX 119 kb)
Additional file 6: Figure S5. Differential abundance detection sensitivity with varied library sizes that are approximately even on average between groups. Label the same as Fig. 4, but with more effect sizes. (PDF 44 kb)
Additional file 7: Figure S6. Differential abundance detection false discovery rate with varied library sizes that are approximately even on average between groups. An expanded Fig. 5. (PDF 40 kb)
Additional file 8: Figure S7. Pseudocount addition to avoid zero increases FDR. The same data as Fig. 7. **a** Uneven library sizes, FDR *p* < 0.05. **b** Uneven library sizes, FDR *p* < 0.01. Pseudo indicates a pseudocount of one was added to the matrix prior to analysis. (PDF 104 kb)
Additional file 9: Code adapted from McMurdie and Holmes [18] to include additional methods. This code is the basis for Figs. 4 and 5.
Additional file 10: Code adapted from McMurdie and Holmes [18] to include additional methods and compositional effects. This code is the basis for Figure 6.
