# Supplementary material for: Normalization and microbial differential abundance strategies depend upon data characteristics
Source: Microbiome. 2017 Mar 3;5:27. doi: 10.1186/s40168-017-0237-y (PMC5335496; doi:10.1186/s40168-017-0237-y)
Supplement: Additional file 1: Figure S1. — Comparison of common distance metrics and normalization methods when low-coverage samples are excluded. The right axis represents the median library size (NL), while the x-axis effect size is the multinomial mixing proportions of the two classes of samples, ocean and feces. For rarefying, samples below the 15th percentile of library size were dropped from the analysis. See caption for Fig. 2 for further details. (PDF 432 kb) [file 40168_2017_237_MOESM1_ESM.pdf]

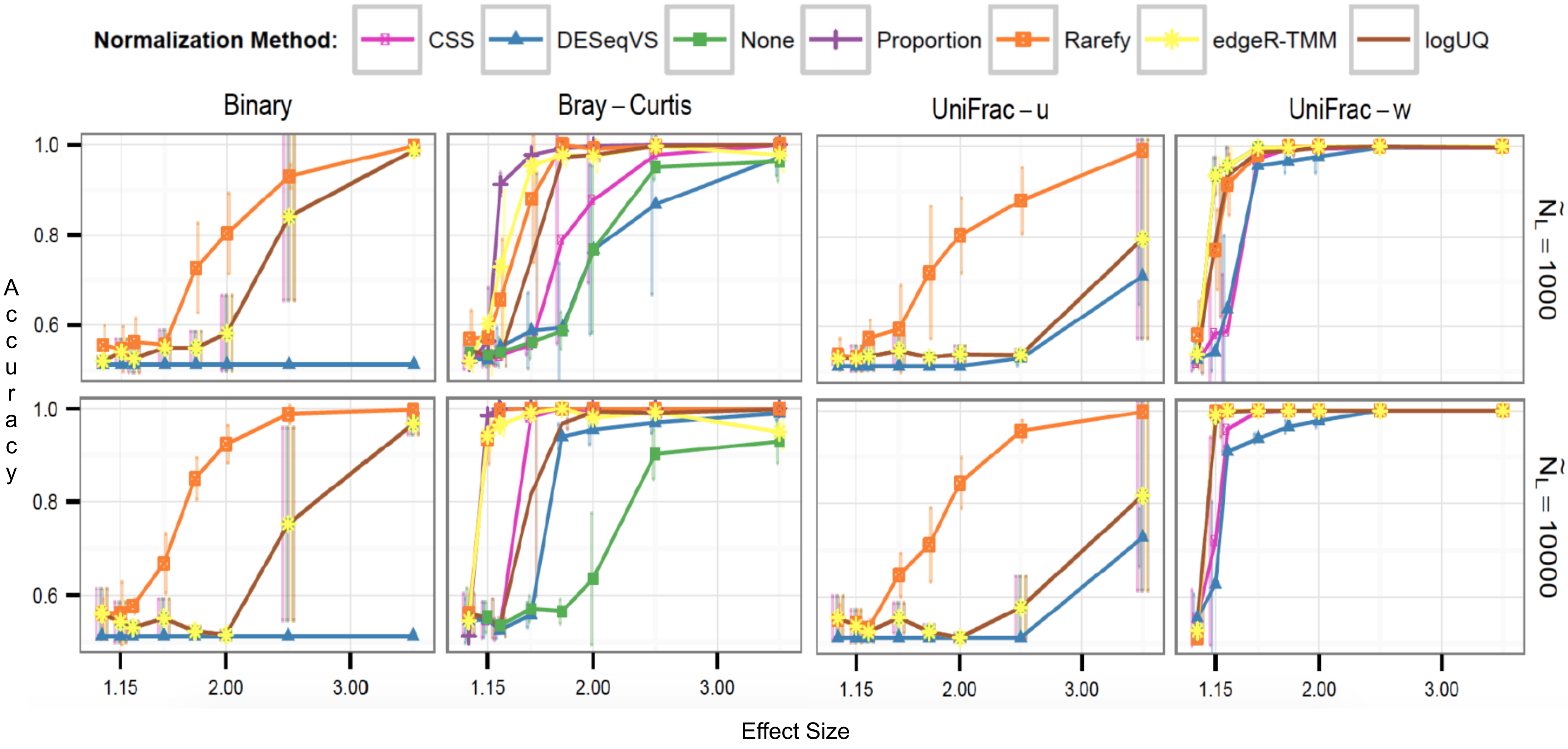

Normalization Method:
CSS
DESeqVS
None
Proportion
Rarefy
edgeR-TMM
logUQ
Binary
Bray – Curtis
UniFrac – u
UniFrac – w
Accuracy
Effect Size
$\tilde{N}_L = 1000$
$\tilde{N}_L = 10000$
