# Supplementary figures and images for: Normalization and microbial differential abundance strategies depend upon data characteristics
Source: Microbiome. 2017 Mar 3;5:27. doi: 10.1186/s40168-017-0237-y (PMC5335496; doi:10.1186/s40168-017-0237-y)

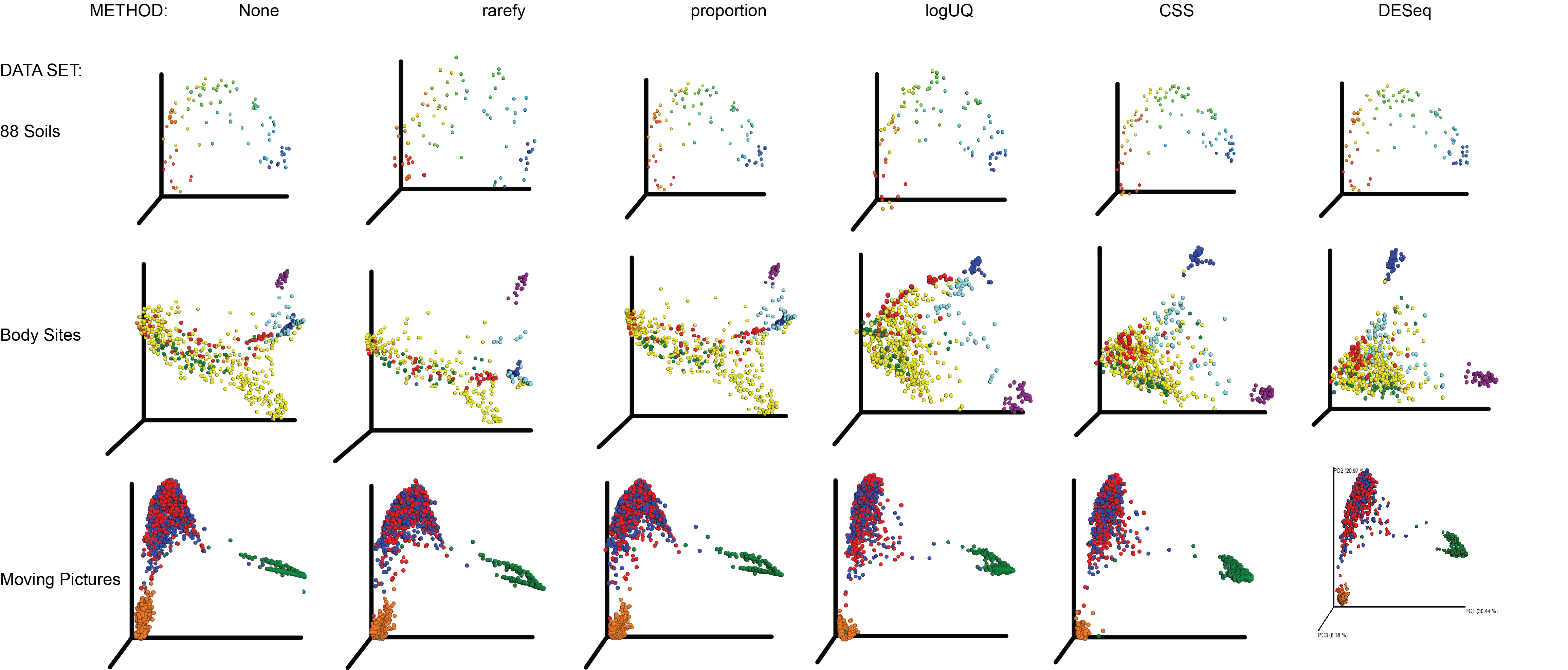

Supplement: Additional file 2: Figure S2. — All normalization techniques on key microbiome datasets, Bray Curtis distance. Rows of panels show (from top to bottom) data from 88 soils [62], body sites [63], and moving pictures [64]. 88 soils are colored according to a color gradient from low to high pH. The Costello et al. body sites’ dataset is colored according to body site feces (blue) and oral cavity (purple); the rest of the colors are external auditory canal, hair, nostril, skin, and urine. Moving pictures dataset: left and right palm (red/blue), tongue (green), and feces (orange). It is important to note that all the samples in these datasets are approximately the same depth, and there are very strong driving gradients. (PNG 1357 kb) [file 40168_2017_237_MOESM2_ESM.png]

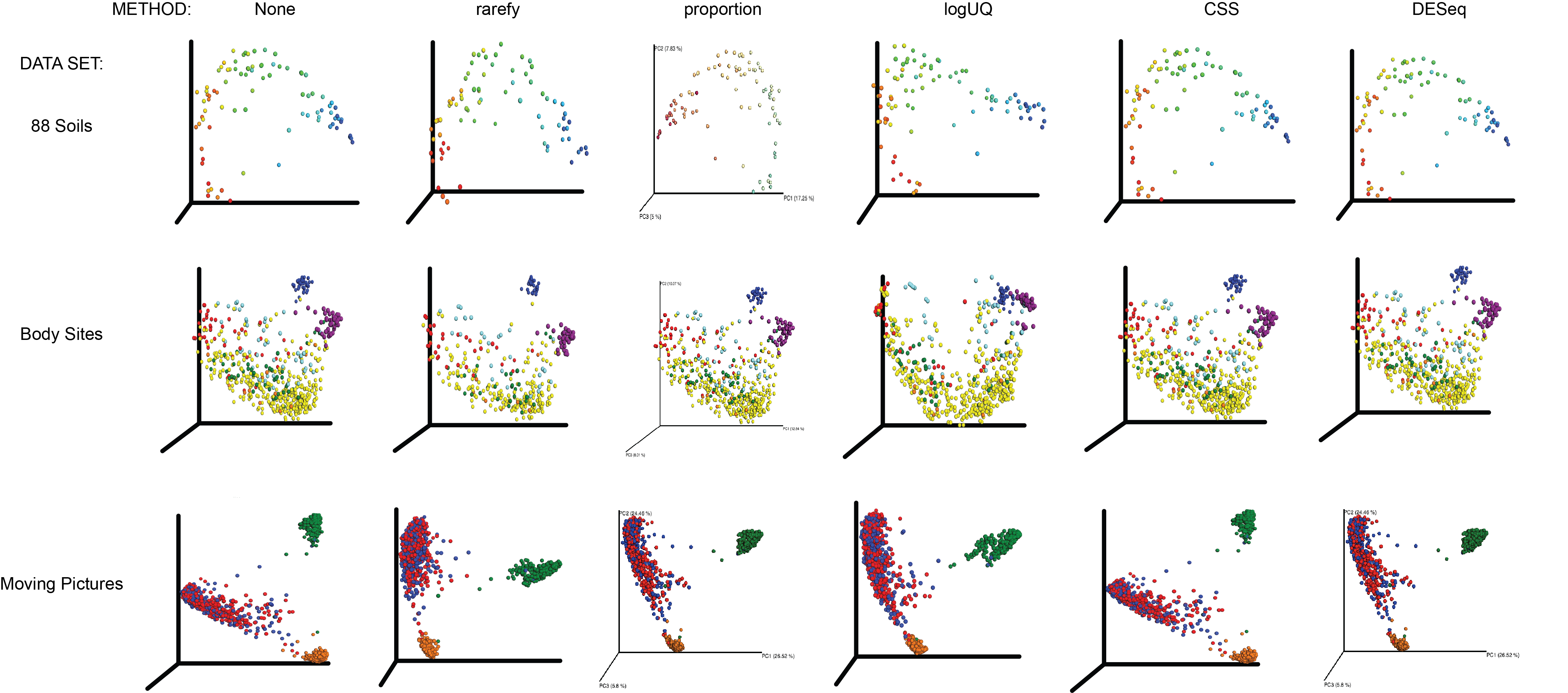

Supplement: Additional file 3: Figure S3. — All normalization techniques on key microbiome datasets, unweighted UniFrac distance. See Figure S3 caption for details. (PNG 1368 kb) [file 40168_2017_237_MOESM3_ESM.png]

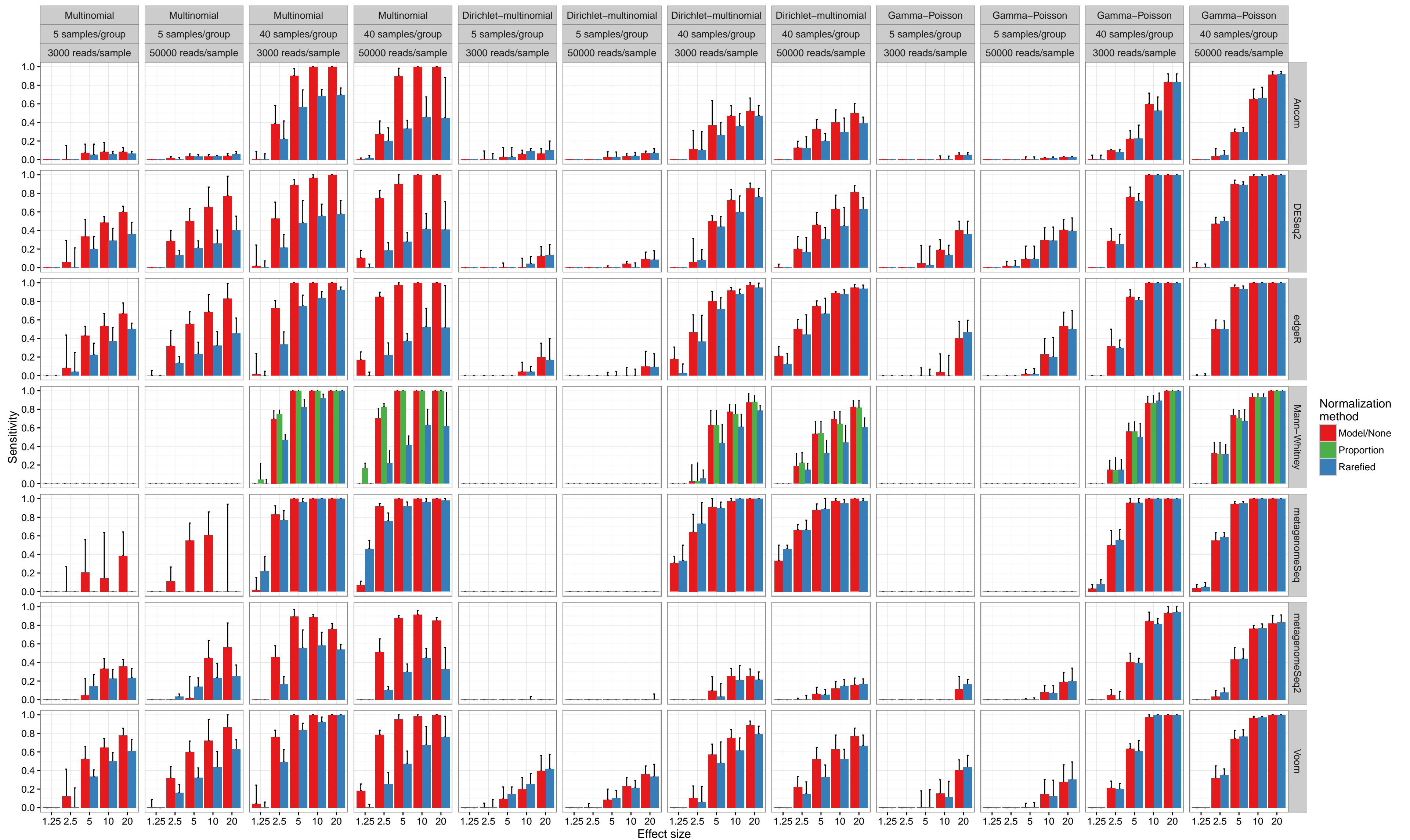

Supplement: Additional file 6: Figure S5. — Differential abundance detection sensitivity with varied library sizes that are approximately even on average between groups. Label the same as Fig. 4, but with more effect sizes. (PDF 44 kb) [file 40168_2017_237_MOESM6_ESM.pdf]

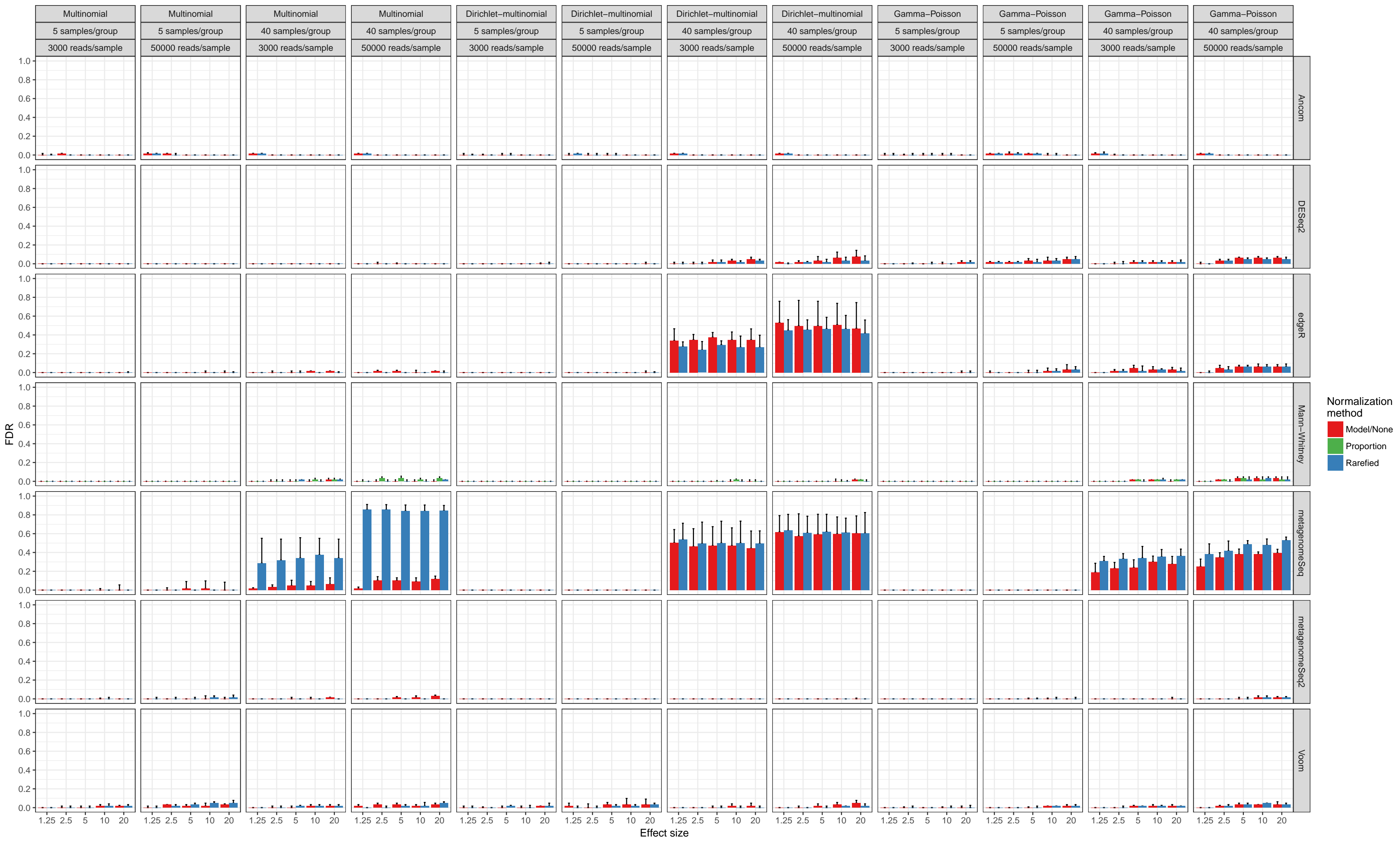

Supplement: Additional file 7: Figure S6. — Differential abundance detection false discovery rate with varied library sizes that are approximately even on average between groups. An expanded Fig. 5. (PDF 40 kb) [file 40168_2017_237_MOESM7_ESM.pdf]

a

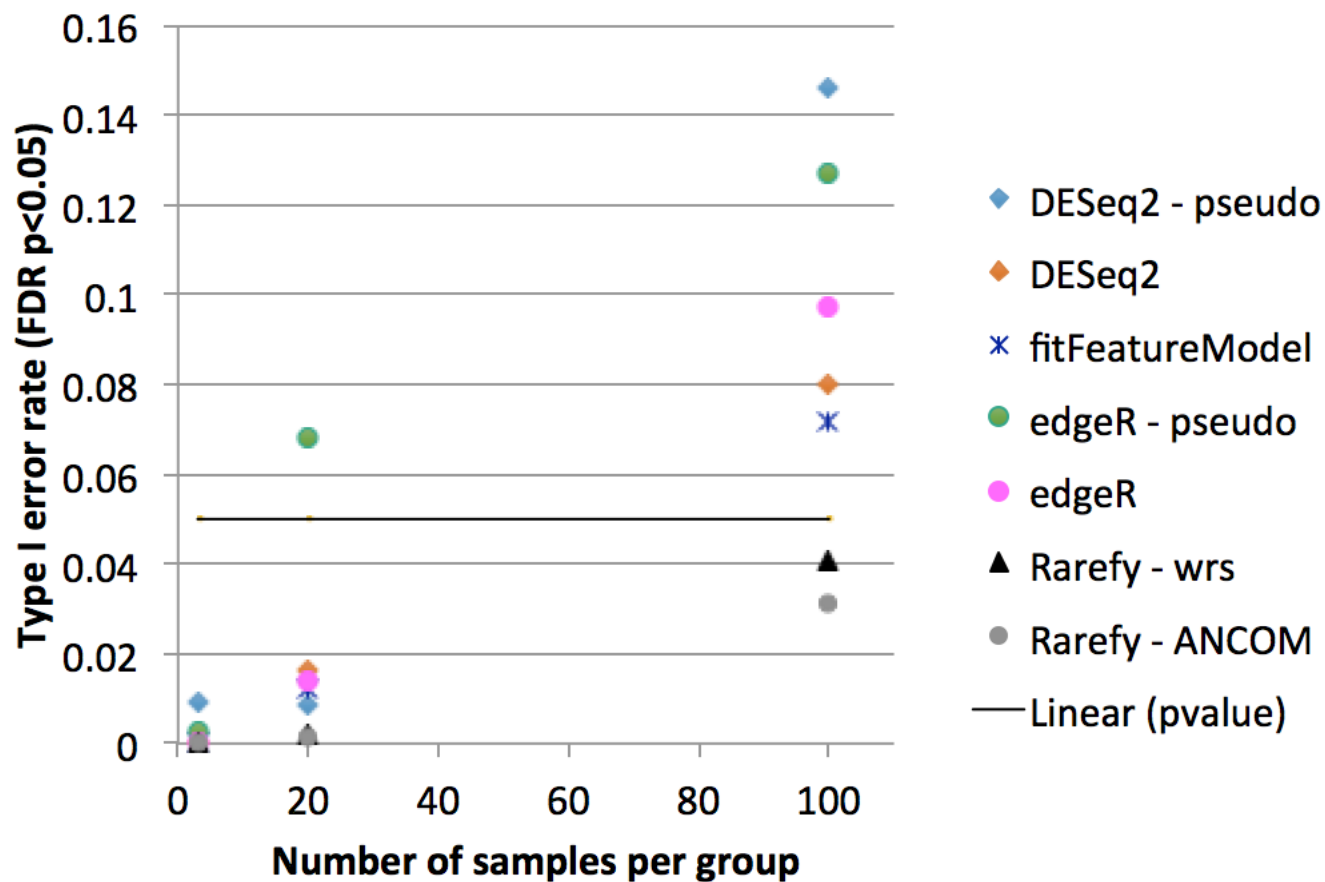

b

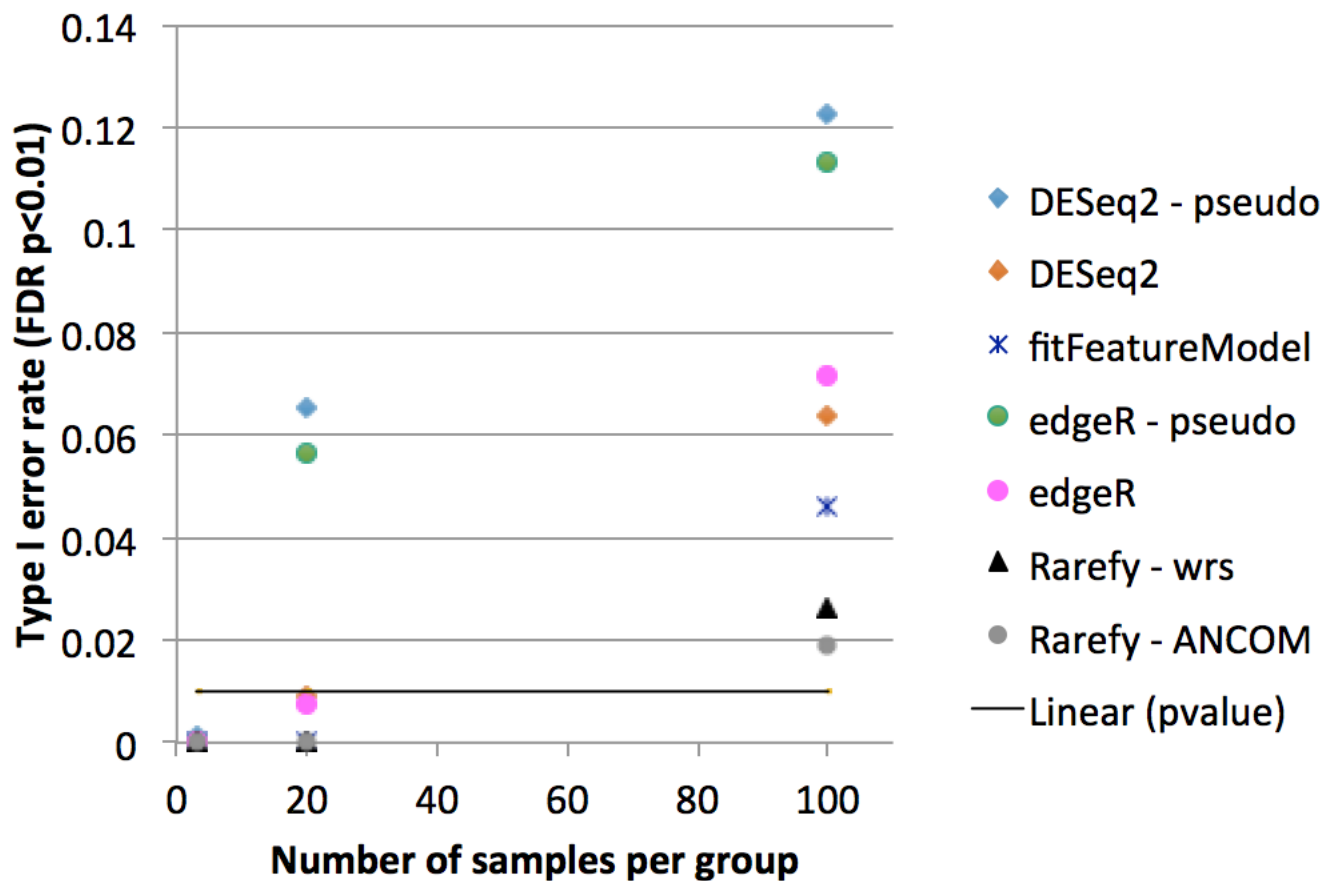

Supplement: Additional file 8: Figure S7. — Pseudocount addition to avoid zero increases FDR. The same data as Fig. 7. a Uneven library sizes, FDR p < 0.05. b Uneven library sizes, FDR p < 0.01. Pseudo indicates a pseudocount of one was added to the matrix prior to analysis. (PDF 104 kb) [file 40168_2017_237_MOESM8_ESM.pdf]
