# Supplementary material for: Normalization and microbial differential abundance strategies depend upon data characteristics
Source: Microbiome. 2017 Mar 3;5:27. doi: 10.1186/s40168-017-0237-y (PMC5335496; doi:10.1186/s40168-017-0237-y)
Supplement: Additional file 4: Figure S4. — Simple example of the reasoning behind differential abundance simulations. a In actual OTU tables generated from sequencing data, the counts (left column) are already compositional and therefore only relative. Application of a constant multiplier to the original multinomial template to create fold-change differences disturbs the distinction between true positive (TP) and true negative (TN) OTUs in the original simulation, but not the balanced simulation. b Creation of compositional data from the multinomial template. The sum constraint that occurs when a researcher samples from the environment can cause, e.g., unchanged OTUs to appear changed in an OTU table. (PDF 199 kb) [file 40168_2017_237_MOESM4_ESM.pdf]

a

Multinomial Template:

OTU Table:

	Ocean_1	Ocean_2
OTU1	5	5
OTU2	11	12
OTU3	40	40

Relative Abundance Data:

	Ocean_1	Ocean_2	Ocean_1/Ocean_2
OTU1	0.09	0.09	1.02
OTU2	0.20	0.21	0.93
OTU3	0.71	0.70	1.02

Original Approach: constant (20) applied to OTU1

	Ocean_1	Ocean_2
OTU1-TP	100	5
OTU2	11	12
OTU3	40	40

	Ocean_1	Ocean_2	Ocean_1/Ocean_2
OTU1-TP	0.66	0.09	7.55
OTU2-TN?	0.07	0.21	0.35
OTU3-TN?	0.26	0.70	0.38

Balanced Approach: constant (20) applied to OTU1, and OTU1d (d=duplicate)

	Ocean_1	Ocean_2
OTU1-TP	100	5
OTU1d-TP	5	100
OTU2	11	12
OTU3	40	40

	Ocean_1	Ocean_2	Ocean_1/Ocean_2
OTU1-TP	0.64	0.03	20.12
OTU1d-TP	0.03	0.64	0.05
OTU2-TN	0.07	0.07	0.92
OTU3-TN	0.26	0.25	1.00

b

Original Approach:

Multinomial Template/Environment:

	Ocean_1	Ocean_2
OTU1	5	5
OTU2	11	12
OTU3	40	40

sample from multinomial →

OTU table:

	Ocean_1	Ocean_2
OTU1	100	5
OTU2	11	12
OTU3	40	40

Compositional Aproach:

Multinomial Template/Environment:

	Ocean_1	Ocean_2
OTU1	100	5
OTU2	11	12
OTU3	40	40

sample from multinomial →

OTU table:

	Ocean_1	Ocean_2
OTU1	43	5
OTU2	2	12
OTU3	11	40
