# Supplementary material for: Normalization and microbial differential abundance strategies depend upon data characteristics
Source: Microbiome. 2017 Mar 3;5:27. doi: 10.1186/s40168-017-0237-y (PMC5335496; doi:10.1186/s40168-017-0237-y)
Supplement: Additional file 5: — Statistical supplement: statistical details of simulation studies. (DOCX 119 kb) [file 40168_2017_237_MOESM5_ESM.docx]

This supplementary section consists of three subsections that correspond to some of the aspects of the differential abundance simulations presented in this paper and in McMurdie and Holmes (2014). In subsection A we demonstrate that the simulation set-up of McMurdie and Holmes (2014) does not necessarily contain true nulls but all are non-null hypotheses. Therefore, all discoveries made by a procedure are true discoveries (non-nulls), there are no false discoveries. In subsection B we describe our refinement of McMurdie and Holmes (2014) set-up so that the set of hypotheses to be tested includes some true nulls and some true non-nulls. In doing so, we can estimate the false discovery rate of any given method (i.e. among all hypotheses rejected, the expected proportion of null hypotheses rejected). In subsection C, we describe why ANCOM (Mandal *et al*., 2015) has a better control of false discovery rate than any of the other methods considered in this paper when testing hypotheses regarding taxa abundance in two ecosystems (or environments) of unit volume.

1. **(McMurdie and Holmes, 2014 [**[**1**](#_ENREF_1)**]) ‘Original’ Simulation corresponding to Figure S5a**

**Step 1 (Generate samples to form Group 1):** Take the raw data of counts available in one of the environments studied in Caporaso et al. [[2](#_ENREF_2)], e.g. freshwater – creek, replicate 1. Suppose $O=(O_{1},O_{2},...,O_{p})'$is the sum of observed abundances of taxa in all the samples and let$U=\sum_{j=1}^{p} O_{j}$.

- Step 1: Generate multinomial random vector $X_{i}=(X_{i1},X_{i2},...,X_{ip})'$ to represent the taxa counts for the $i$-th sample.
- Steps 1 repeated$n$times to obtain $n$random samples to represent Group 1.

**Step 2 (Generate samples to form Group 2):**

- Step 2(a). As in Step 1, generate multinomial random vector $Z_{i}=(Z_{i1},Z_{i2},...,Z_{ip})'$ to represent the taxa counts for the $i$-th sample. By construction,$X_{i}=(X_{i1},X_{i2},...,X_{ip})'$and $Z_{i}=(Z_{i1},Z_{i2},...,Z_{ip})'$ are identically and independently distributed.
- According to Figure 2B on page 6 of McMurdie and Holmes (2014) [[1](#_ENREF_1)], the OTU counts for a random subset of $r$taxa (e.g. 30 in their paper) is given by$Y_{ij}=m\text{ }\times Z_{ij}$, where $m\text{ }$represents the effect size (e.g., 10) and the rest $p-r$are taken to be$Z_{ij}$. In their paper, they take$r=30.$ To restate more precisely:

$$Y_{ij}=\left\{\begin{matrix} m\text{ }\times Z_{ij}\text{ with probability }\frac{r}{p}\text{ } \\ Z_{ij}\text{ with probability 1-}\frac{r}{p} \end{matrix}\right.$$

**Note :** By construction, for the$i$-th sample and $j$-th taxa,$1\leq j\leq p$, $\begin{matrix} E(Y_{ij})=m\times\frac{r}{p}E(Z_{ij})+\frac{p-r}{p}E(Z_{ij}) \\ =\frac{m\times r+(p-r)}{p}E(Z_{ij})=\frac{m\times r+(p-r)}{p}E(X_{ij}). \end{matrix}$

Unless$m=1$or $r=0$, in general$E(Y_{ij})\neq E(X_{ij}).$ Hence the simulations conducted according to Figure 2B in McMurdie and Holmes (2013) [[1](#_ENREF_1)] there will be no non-differentially abundant taxa by this simulation set up. So it is not surprising that false positive rates are out of control.

1. **‘Balanced Simulation’ corresponding to Figure S5a**

**Step1:** Take the raw data of counts available in freshwater – creek, replicate 1. Suppose $O=(O_{1},O_{2},...,O_{r},O_{r+1},O_{r+2},...,O_{2r},O_{2r+1},O_{2r+2},...,O_{p})'$is the sum of observed abundances of taxa in all the samples, where$(O_{r+1},O_{r+2},...,O_{2r})=(O_{1},O_{2},...,O_{r})'$. We define

Let $P_{i}=\left\{\begin{matrix} m\text{ }\times O_{i}\text{ for }1\leq i\leq r\text{ } \\ O_{i}\text{ for } r+1\leq i\leq p \end{matrix}\right.$

and $Q_{i}=\left\{\begin{matrix} O_{i}\text{ for }1\leq i\leq r\text{ } \\ m\text{ }\times O_{i}\text{ for } r+1\leq i\leq2r \\ O_{i}\text{ for }2r+1\leq i\leq p\text{ } \end{matrix}\right.$

Note that$U=\sum_{j=1}^{p} P_{j}=\sum_{j=1}^{p} Q_{j}$. The relative abundance of the$j$-th taxa in Group 1 is given by$s_{j}=\frac{P_{j}}{U},j=1,2,...,p,$ and in Group 2 it is given by$r_{j}=\frac{Q_{j}}{U},j=1,2,...,p.$

**Step 2(a)** **Generation of Group 1 samples:** Generate a random library size$S_{i}$ for the $i$-th sample from the collection of libraries in Caporaso *et al.* [[2](#_ENREF_2)]. Then, using the relative abundance vector$(s_{1},s_{2},...,s_{p})'$, generate multinomial random vector $X_{i}=(X_{i1},X_{i2},...,X_{ir},X_{ir+1},X_{ir+2},...,X_{i2r},X_{i2r+1},X_{i2r+2},...,X_{ip})'$ to represent the taxa counts for the $i$-th sample in Group 1. Similarly, by generating a random library size$S_{i}$ for the $i$-th sample from the collection of libraries in Caporaso *et al.* [[2](#_ENREF_2)] and using the relative abundance vector$(r_{1},r_{2},...,r_{p})'$, we generate multinomial random vector $Y_{i}=(Y_{i1},Y_{i2},...,Y_{ir},Y_{ir+1},Y_{ir+2},...,Y_{i2r},Y_{i2r+1},Y_{i2r+2},...,Y_{ip})'$ to represent the taxa counts for the $i$-th sample in Group 2.

Observe that

$$E(X_{ij})=s_{j}E(S_{i})=\left\{\begin{matrix} \frac{m\times O_{j}}{U}E(S_{i})\text{ }1\leq j\leq r \\ \frac{O_{j}}{U}E(S_{i})\text{ } r+1\leq j\leq p \end{matrix}\right.$$

and

$$E(Y_{ij})=r_{j}E(S_{i})=\left\{\begin{matrix} \frac{O_{j}}{U}E(S_{i})\text{ }1\leq j\leq r \\ \frac{m\times O_{j}}{U}E(S_{i})\text{ } r+1\leq j\leq2r \\ \frac{O_{j}}{U}E(S_{i})\text{ } 2r+1\leq j\leq p \end{matrix}\right.$$

Hence we have

$$E(X_{ij}-Y_{ij})=\left\{\begin{matrix} \frac{(m-1)\times O_{j}}{U}E(S_{i})\text{ }1\leq j\leq r \\ \frac{(1-m)\times O_{j}}{U}E(S_{i})\text{ } r+1\leq j\leq2r \\ 0\text{ } 2r+1\leq j\leq p \end{matrix}\right.$$

Thus first $2r$components are true positives because$E(X_{ij}-Y_{ij})\neq0$ for $1\leq j\leq2r$ and the last $p-2r$are true negatives because $E(X_{ij}-Y_{ij})=0$ for$2r+1\leq j\leq p$.

1. **Inference regarding taxa abundance: Figure S5b**

Suppose $\mu_{X_{i}}$and$\mu_{Y_{i}}$denote the true abundance of the$i^{th}$taxon,$i=1,2,...,p$, in the environments A and B respectively. Suppose$\mu_{X_{1}}\neq\mu_{Y_{1}}$ , but $\mu_{X_{i}}=\mu_{Y_{i}}$for all$i=2,...,p$. Thus, taxon 1 is true positive and rest of the taxa, $i=2,...,p$, are true negatives in the two environments A and B.

For, $i=1,2,...,p$, let $\theta_{i}=\frac{\mu_{X_{i}}}{\sum_{j=1}^{p} \mu_{X_{j}}}$and$\eta_{i}=\frac{\mu_{Y_{i}}}{\sum_{i=1}^{p} \mu_{Y_{i}}}$. Suppose a researcher draws samples from a multinomial distribution with probability vectors $\theta=(\theta_{1},\theta_{2},....,\theta_{p})'$and$\eta=(\eta_{1},\eta_{2},....,\eta_{p})'$

then note that, for$i=2,...,p$, $\theta_{i}\neq\eta_{i}$, even though$\mu_{X_{i}}=\mu_{Y_{i}}$.

Among all the methods discussed in the paper, ANCOM (Mandal *et al.,* 2015) is the only method that is designed for testing the null hypotheses$H_{0}:\mu_{X_{i}}=\mu_{Y_{i}},i=1,2,...,p$ and hence is expected to provide a better control of false discovery rate than any of the other methods because none of the other methods described in this paper are designed to test hypotheses regarding the actual parameters of interest, namely, $\mu_{X_{i}}$and$\mu_{Y_{i}}$. Consequently, they are expected to have inflated false discovery rates. Previously, Mandal *et al.* (2015) demonstrated the FDR control of ANCOM using a Poisson-Gamma simulation set-up and we now duplicate those results for multinomial data [3].

**References**

1. McMurdie PJ, Holmes S. Waste Not, Want Not: Why Rarefying Microbiome Data Is Inadmissible. PLoS computational biology. 2014;10(4). doi 10.1371/Journal.Pcbi.1003531.

2. Caporaso JG, Lauber CL, Walters WA, Berg-Lyons D, Lozupone CA, Turnbaugh PJ et al. Global patterns of 16S rRNA diversity at a depth of millions of sequences per sample. Proceedings of the National Academy of Sciences of the United States of America. 2011;108 Suppl 1:4516-22. doi:10.1073/pnas.1000080107.

3. Mandal S, Van Treuren W, White RA, Eggesbø M, Knight R, Peddada SD (2015). Analysis of composition of microbiomes: a novel method for studying microbial composition. Microbial Ecology in Health and Disease, 26, 1 – 7.
